# Enhancing Veterinary Education: Integrating Wildlife Medicine Into Undergraduate Training in Kenya

**DOI:** 10.1155/vmi/4418942

**Published:** 2026-07-03

**Authors:** Grace Watene, Dale Smith, Tequiero Abuom, Ambrose Kipyegon, Isaac Lekolool, Ruth Omani, Magda Scarlett

**Affiliations:** ^1^ Veterinarians International, New York, 10001, New York, USA; ^2^ Ontario Veterinary College, University of Guelph, Guelph, Canada, uoguelph.ca; ^3^ Department of Clinical Studies, University of Nairobi, Nairobi, Kenya, uonbi.ac.ke; ^4^ Department of Veterinary and Capture Services, Kenya Wildlife Service, P.O Box 40241-00100, Nairobi, Kenya, kws.go.ke; ^5^ Faculty of Veterinary Medicine, University of Liège, B-4000, Liège, Belgium, ulg.ac.be

## Abstract

Veterinary education has traditionally focused on domestic animals, yet the expanding role of veterinarians in wildlife health necessitates the integration of wildlife medicine into undergraduate training. In Kenya, despite the ecological and economic importance of wildlife, veterinary curricula provide limited structured exposure to wildlife medicine. This study aimed to (i) identify gaps in undergraduate veterinary training related to wildlife health and (ii) use these findings to inform the design of a stakeholder‐driven pilot training model. A mixed‐methods approach was applied using two structured surveys administered to veterinary students and graduates (*n* = 83) and employers (*n* = 33). Quantitative data were analyzed using descriptive statistics, while qualitative responses were examined through a thematic analysis. The findings were further refined through a stakeholder validation workshop involving academia, wildlife practitioners, and conservation stakeholders. While 71% of respondents expressed interest in wildlife health, only 22% felt adequately prepared for practice. Key gaps included insufficient curriculum content, limited time allocation, and lack of practical exposure. Employers reported variability in graduate competence, reinforcing the need for improved training. These findings informed the development of a 10‐module pilot training program combining theoretical instruction with field‐based experiential learning. The study highlights the feasibility of a research‐informed, stakeholder‐driven approach to curriculum development in wildlife medicine. However, the effectiveness of the intervention was not evaluated, and further longitudinal research is required to assess its impact on competencies, career outcomes, and workforce preparedness in wildlife health.

## 1. Introduction

Today, increasing numbers of veterinarians are working with nondomestic species; however, despite this trend, education in wildlife medicine has not been fully integrated into formal veterinary training at the undergraduate level [1, 2]. In the past, the primary role of veterinarians was the intervention and management of domestic populations experiencing a health crisis, but recently, there has been increasing interest in ensuring the health and well‐being of wildlife populations. The initial interest in wildlife populations was to protect humans from zoonotic menaces such as rabies, but due to the economic role of wildlife, this interest has expanded to include wildlife health, breeding programs, and conservation [3]. The role of veterinarians in wildlife management has become even more important recently with the emergence of infectious diseases from wild animal environments. SARS‐CoV‐2, monkeypox, MERS‐CoV, and Ebola are examples of infectious diseases that have spread recently into the human population and have had high socioeconomic impacts on global health and world economies [4, 5]. Shared infections at the livestock–wildlife–human interface underscore the interconnectedness of wildlife, human, and livestock ecosystems and frequently impact multiple facets of public health, economy, and biodiversity [6].

With this increase in demand for veterinary oversight in wildlife management comes the obligation of veterinarians to become better capacitated, particularly on the physiology, anatomy, behavior, nutrition, and diseases of wildlife [3, 7]. With the increasing role of veterinarians in supporting the welfare and health of humans and the environment as well as domestic and wild animals, there is an active need for undergraduate programs to evolve and encompass this new frontier [7]. This is more so in low‐ and middle‐income countries such as Kenya where the lives of humans, domestic animals, and wild animals are increasingly interconnected. An example of this is major water towers that are part of the protected wildlife inhabited areas [8, 9]; however, the undergraduate training programs in veterinary medicine [10] do not include aspects of wildlife training. While these gaps have been recognized globally, there is limited context‐specific evidence from Kenya incorporating perspectives from students, graduates, and employers, and limited application of such evidence to inform curriculum design.

This paper identified gaps in veterinary training that hinder Kenya’s veterinarians from fulfilling the demands of the wildlife sector and used these findings to inform the design of a stakeholder‐driven pilot training model. The study did not evaluate the effectiveness of intervention. This contributes to the enhancement of practical knowledge of wild animal health and a broad perspective on ecosystem health. This is increasingly crucial as zoonotic cases surge and global health issues continue to become more complex. It is particularly important in the Kenyan context where wildlife has sociocultural, economic, and esthetic values to the Kenyan people and their economy.

## 2. Methodology

### 2.1. Online Surveys and Stakeholder Validation Meeting

Two surveys were conducted simultaneously. The first targeted graduates of the veterinary program in Kenya (alumni) and current students (*n* = 83) from and at the University of Nairobi. They were interviewed using an online questionnaire (20 questions) that included both open‐ and closed‐ended questions to obtain their views regarding the strengths, weaknesses, opportunities, and challenges in the Bachelor of Veterinary Medicine (BVM) program as relates to the veterinary care of free‐ranging and captive wild animals. The questionnaire was developed based on a review of relevant literature and consultation with subject matter experts and was designed to capture both quantitative responses and qualitative insights.

The second survey of 16 questions was sent to targeted employers of veterinary graduates in the wildlife industry and educational institutions (*n* = 33). Employers were purposively selected to represent key sectors involved in wildlife health, including conservation organizations, academia, and veterinary services. The two online surveys were conducted in November 2022. Participation was voluntary, and respondents were recruited through institutional networks and professional contacts.

Data were analyzed using SPSS. Descriptive statistics (frequencies and proportions) were used to summarize quantitative data, while qualitative responses were reviewed and grouped into themes using a thematic analysis approach. The resultant report was discussed and validated in a stakeholder workshop with pertinent stakeholders (e.g., wildlife conservancy managers, Kenya Wildlife Service veterinarians, and academia). The workshop functioned as a consultative validation exercise, where survey findings were presented and discussed to reach consensus on key gaps and priority areas for curriculum development.

At the end of the workshop, an intervention was developed for implementation where a draft list of modules to be developed was established, the experts to develop these modules were identified, and it was agreed that these modules would be taught to 4th‐year students as an extracurricular program during their free time in order to supplement the current wildlife medicine curriculum. The development of the intervention was therefore directly informed by survey findings and stakeholder input, representing a research‐informed, stakeholder‐driven design process.

## 3. Results

### 3.1. Veterinary Graduates and Current Veterinary Students

There was a response rate of 88% (*n* = 83). The majority (69%) of the respondents were current final‐year veterinary medicine students (as of November 2022), while 31% were practicing veterinarians in Kenya. Regarding prior interest in working with free‐ranging and captive wild animals, the majority (71%) of respondents indicated that they had this interest when they joined the veterinary program as undergraduates, suggesting that interest in wildlife health exists prior to formal training. Among those interested in wild animal health before joining veterinary school, 38% reported a passion for wild animal work, 19% wanted to contribute to conservation efforts, 8% sought experience in wild animal behavior, and 6% were motivated by employment opportunities.

### 3.2. Employers of Veterinary Graduates

The employer respondents (*n* = 33) were drawn from wildlife conservancies, Kenya Wildlife Service, academia, and the private sector. Half of the respondents reported that veterinary graduates were competent to handle wild animal health upon employment, while the other 50% indicated that new veterinary graduates were not adequately prepared. This split perception highlights variability in graduate preparedness and reinforces the need for improved training in wildlife medicine.

### 3.3. Qualitative Findings

Qualitative responses from veterinary students and recent graduates were analyzed thematically to explore interest in wild animal health and perceptions of training adequacy. The key themes are presented in Tables 1 and 2.

**TABLE 1:** Thematic analysis of interest in wild animal health among students and graduates.

Theme	Example quotes
Early passion for wildlife	“I have always been passionate about wildlife from a young age and visited the orphanage several times as a child.”

Inspiration from media and personal experiences	“Growing up, I read a lot of National Geographic magazines and anything with wild animals captivated me. The first time I visited a national park, I realized I would find fulfillment working with wild animals.”
	“I have always been fascinated by wildlife. I have watched National Geographic ever since I was a kid. Getting an opportunity to interact with them as a veterinarian will be an honour.”

Influence of training and field exposure	“We were introduced to wildlife diseases and immobilization courses, and I managed to go for industrial attachment at KWS in my first and 4th year.”
	“The field exposure program and a trip to Ol‐Jogi during my final year was a game changer.”

Limited but stimulating curriculum exposure	“The theoretical knowledge was focused on domesticated animals and occasionally, especially with regards to zoonotic diseases, would wildlife be mentioned.”
	“Though it was not much, the content we were given sparked my interest to learn more about wildlife.”

Lack of prior exposure	“Where I come from this is not an area that’s well known. Mostly we only look at veterinary medicine as clinical practice.”
	“I was never exposed to such an area prior.”

**TABLE 2:** Thematic analysis of perceptions of adequacy of veterinary training for wild animal practice.

Theme	Example quotes
Inadequate emphasis on wildlife practice	“There is very little emphasis on wild animal practice during teaching.”
Comprehensive coverage (minority view)	“The course covers a lot of components in wildlife medicine from restraint, diseases, and treatment to surgeries and wildlife‐human interactions.”
Insufficient training	“There isn’t enough training with regards to wild animals yet it’s the same vets who take care of their welfare.”
Lack of practical exposure	“Less information is taught on medication used in wild animals and no exposure to wildlife medicine practicals.”

While respondents demonstrated strong intrinsic and experiential drivers of interest in wildlife health, including early exposure, media influence, and limited but meaningful training experiences, a subset of respondents highlighted a lack of exposure as a key reason for limited interest. In addition to factors influencing interest in wildlife health, respondents also reflected on the adequacy of their training for wild animal practice. Only 22% of respondents indicated that the veterinary course adequately equipped them for wild animal practice, while 78% reported that the current structure did not sufficiently cover relevant aspects. Qualitative responses explaining these perceptions are summarized in Table 2.

**FIGURE 1:**
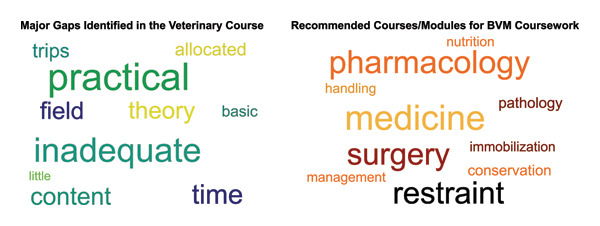
Identified inadequacies and recommended courses/modules in the veterinary course.

The qualitative findings highlight that perceived inadequacies in training were primarily attributed to insufficient time allocation, limited depth of content, and a lack of practical and field‐based exposure. These findings align with the quantitative results and reinforce the need for enhanced curriculum integration of wildlife medicine. The specific areas identified as contributing to inadequacies in the current veterinary course included inadequate time allocation, shallow or insufficient content, and a lack of practical and fieldwork sessions (Figure 1). When asked what courses or modules should be included in the BVM curriculum for those interested in wild animal practice, respondents most frequently recommended increased focus on wildlife medicine and animal restraint.

**FIGURE 2:**
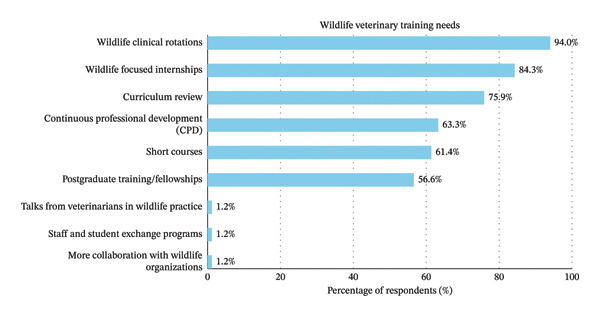
Suggestions on how to address gaps in veterinary training.

Respondents also provided recommendations on how to address these gaps (Figure 2). Key suggestions included dedicated wildlife clinical rotations (94%), wildlife‐focused internships (84.3%), and comprehensive curriculum review to incorporate more wildlife content (75.9%). Additional recommendations included ongoing Continuous Professional Development (CPD) programs (63.9%), short, specialized courses in wildlife medicine (61.4%), and postgraduate training opportunities (56.6%). Less frequently mentioned suggestions included increased collaboration with wildlife organizations, staff and student exchange programs, and guest lectures from experienced wildlife veterinarians.

### 3.4. Intervention Design and Implementation (Pilot)

To address the gaps identified through the survey and stakeholder validation process, a 10‐module pilot training program was developed in September 2023 through consultative discussions involving stakeholders and subject matter experts using a One Health approach. The development of the program was directly informed by the findings of the gap analysis and subsequent stakeholder engagement. The modules covered key areas relevant to wildlife health and management, including management, human–wildlife conflict, immobilization, rescue protocols, neonatal care, forensics, survival techniques, ecosystem health, conservation, translocation, and rehabilitation. The training was offered to fourth‐year veterinary students from the two veterinary training institutions in Kenya, the University of Nairobi and Egerton University, as an extracurricular program designed to complement existing curricula.

**FIGURE 3:**
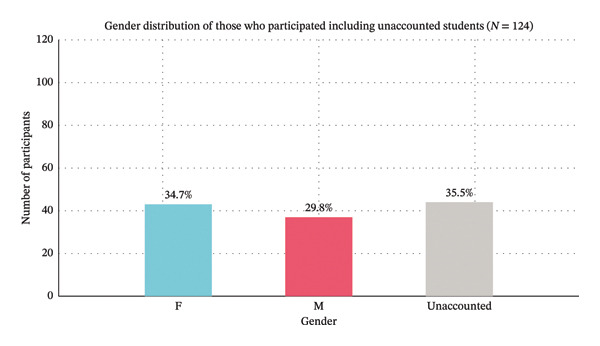
Gender distribution of those who participated in the theoretical component of the course.

Eighty out of 124 eligible students (65%) enrolled in the theoretical component of the program. A slightly higher proportion of female students enrolled compared to male students (34.7% vs. 29.8%, respectively) (Figure 3).

**FIGURE 4:**
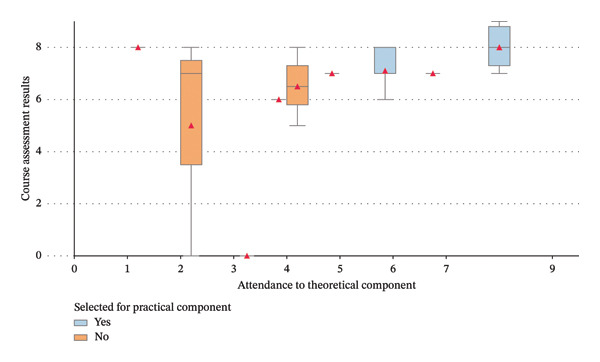
Course assessment results across attendance levels (with practical component selection).

A subset of trainees (*n* = 20; 16.1%) was selected to participate in a 1‐week field‐based practical training program conducted in June 2024 at wildlife conservancies in Laikipia, Kenya. Due to resource constraints, participation in the practical component was limited. Selection was based on attendance and performance in the theoretical component, with priority given to students demonstrating higher engagement and assessment scores (Figure 4).

**FIGURE 5:**
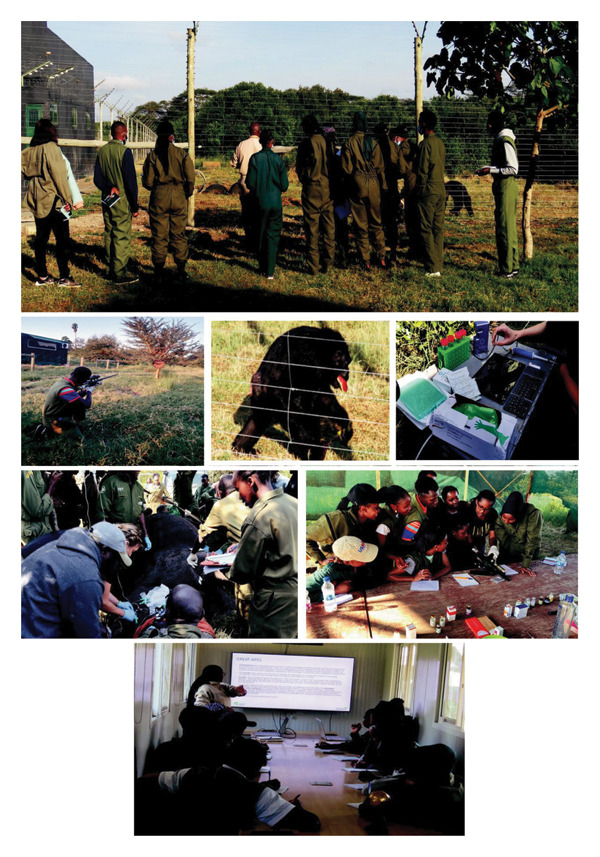
Students participating in field‐based wildlife training activities at Ol Pejeta and Loisaba Conservancies (captured by Grace Watene).

The field‐based training component provided exposure to practical aspects of wildlife health and conservation, including wildlife immobilization, sanctuary animal management, and ecosystem health at the wildlife–livestock–human interface (see Figure 5). Training activities included demonstrations, guided field sessions, and interactive discussions facilitated by experienced wildlife veterinarians from national institutions, including the Kenya Wildlife Service, as well as international collaborators. It is important to note that this pilot intervention was not designed to evaluate training outcomes or effectiveness. Rather, it represents a feasibility‐based, stakeholder‐informed model for integrating wildlife medicine into undergraduate veterinary training. Further longitudinal evaluation is required to assess its impact on student competencies and career trajectories.

## 4. Discussion

There is growing recognition of the critical role veterinarians play in wildlife management, particularly within interconnected human, animal, and environmental systems. In the Kenyan context, where these interfaces are especially pronounced, strengthening veterinary capacity in wildlife health is increasingly important. This study identifies gaps in veterinary training and describes a stakeholder‐informed pilot approach to addressing these gaps.

The findings of the surveys indicate the presence of gaps in Kenyan veterinary training with respect to developing competence in wildlife medicine and management. Fifty percent of employer respondents reported that graduates were competent, while the remaining 50% indicated that graduates were not adequately prepared and, in some cases, required additional training outside the country. This finding has been previously documented [1, 2, 11] and it is well recognized that existing veterinary curricula are geared toward domestic animal health [12, 13]. This is not surprising as the greatest needs and opportunities have been historically and economically in domestic animal medicine [14]. Most of the respondents (75%) agreed that the materials provided during the BVM training stimulated interest in working with wild animals but did not offer sufficient practical experience to support exploring a career in wildlife health. This is despite the respondents (78%) indicating that they had a prior interest in working with free‐ranging and captive wild animals. Together, these findings suggest that while the curriculum is effective in stimulating interest, it does not adequately support the development of practical competence required for wildlife health practice [15, 16]. This gap between interest and preparedness highlights a need to strengthen the applied components of training rather than content alone, particularly through experiential learning approaches [13]. This disconnect is further reinforced by employer perspectives, suggesting that the gap is not only perceived by students but also reflected in workforce preparedness. Collectively, these findings point to a structural limitation within existing curricula, where exposure to wildlife medicine is present but not sufficiently integrated, applied, or sustained to support competency development [1, 11].

The recommendations received in these surveys align with the literature emphasizing the importance of hands‐on experience and curriculum improvements in veterinary education [1, 11]. Moreover, integrating interdisciplinary approaches and partnerships with wildlife [15, 16] can further enhance practical exposure and education in wildlife health. In this context, the findings suggest that curriculum reform should prioritize structured and sustained exposure to wildlife systems, rather than isolated or ad hoc learning opportunities [1, 11].

The combination of theoretical training and practical exposure represents a stakeholder‐informed approach to addressing gaps in wildlife practice training [16, 17]. By integrating interdisciplinary knowledge with hands‐on experience, the program provides a potential model for enhancing veterinary education in wildlife health. However, its impact on student competencies and career outcomes was not evaluated within this study. Participation in the practical component was limited due to resource constraints, with only a small proportion of students able to take part. This limitation is important to consider, as restricted access to experiential training may reinforce disparities in learning opportunities and competency development [18]. In addition, the long‐term impacts of the intervention remain unknown, as participants have yet to complete their training and transition into the workforce. Nevertheless, this pilot provides a foundation for future work, including longitudinal tracking of student competencies and career trajectories to better understand the impact of such interventions on workforce preparedness in wildlife health.

## 5. Conclusion

Addressing gaps in veterinary training for wildlife health requires a multifaceted approach that integrates theoretical instruction with practical exposure. This study identified key gaps in undergraduate veterinary training in Kenya and used these findings to inform the design of a stakeholder‐driven pilot training model. While the intervention demonstrates the feasibility of a research‐informed approach to curriculum enhancement, its effectiveness has not yet been evaluated. Further longitudinal assessment is required to determine its impact on student competencies, workforce preparedness, and career trajectories in wildlife health.

## Author Contributions

Conceptualization: Grace Watene, Dale Smith, Tequiero Abuom, Ambrose Kipyegon, and Magda Scarlett. Methodology: Grace Watene, Dale Smith, Tequiero Abuom, Ambrose Kipyegon, Isaac Lekolool, and Magda Scarlett. Formal analysis, writing, reviewing, and editing: Grace Watene, Dale Smith, Tequiero Abuom, Ambrose Kipyegon, Isaac Lekolool, Ruth Omani, and Magda Scarlett.

## Funding

This study was supported by te Veterinarians International through internal programmatic funding. No specific grant number applies.

## Disclosure

All photographs, illustrations, and graphical elements used in this manuscript were captured or created by the authors. No third‐party or stock imagery was used in the preparation of this article. Figures were synthesized using original photographs, data visualizations, and design components generated specifically for this study. All authors have read and agreed to the published version of the manuscript. Elements of this work were presented in abstract form at the 8th World One Health Congress (2024).

## Ethics Statement

This research involved low‐risk, anonymous surveys and educational stakeholder workshops with adult participants. Based on prevailing institutional and national guidelines, formal ethics committee review was not required. Nonetheless, the study was conducted in full accordance with ethical research standards, including the use of informed written consent and safeguarding participant anonymity.

All participants in the surveys and stakeholder validation workshop provided written informed consent before their involvement in the study. Participation was entirely voluntary, with respondents informed of the purpose of the study, their rights, and their freedom to withdraw at any time. In addition, written consent for the publication of identifiable photographs was obtained from all individuals appearing in images included in this manuscript. No sensitive personal or clinical data were collected.

## Conflicts of Interest

The authors declare that some of the authors are employees of Veterinarians International. The research and publication were conducted as part of their professional duties. The remaining authors declare no conflicts of interest.

## Data Availability

All data related to this study are available upon reasonable request from the corresponding author, Grace Watene, with datasets maintained by Veterinarians International in collaboration with the University of Nairobi.
